# Leveraging drug-specific genes to identify sensitizers for resistant cancer cell lines

**DOI:** 10.1038/s41420-026-03033-x

**Published:** 2026-04-07

**Authors:** G. Pepe, E. Valentini, R. Appierdo, C. Pontecorvi, L. Parca, G. Ausiello, S. Galardi, M. Helmer-Citterich, PF Gherardini

**Affiliations:** 1 Department of Biology, University of Rome Tor Vergata, Rome, Italy; 2 PhD Program in Cellular and Molecular Biology, Department of Biology, University of Rome “Tor Vergata”, Rome, Italy; 3 Present Address: Italian Space Agency, Rome, Italy

**Keywords:** Cancer therapeutic resistance, Molecular biology

## Abstract

Therapeutic resistance remains a major obstacle in oncology, often arising from transcriptional reprogramming that enables cancer cells to escape drug-induced cytotoxicity. We aimed to develop a computational-experimental strategy to identify compounds capable of reversing resistance phenotypes. We integrated previously defined Drug-Specific Genes (DSGs), expression markers of drug sensitivity or resistance, with perturbational profiles from the Connectivity Map (CMap). Candidate compounds were prioritized based on their predicted ability to shift DSG expression toward a sensitized state. The top-ranked compound was validated in resistant HeLa and NCI-H1299 cell lines using BMS-345541 and Vorinostat as primary agents. Cell viability, apoptosis, and cell cycle progression were assessed. Chaetocin consistently emerged as a leading sensitizer in silico. Experimental validation confirmed that chaetocin enhanced the activity of BMS-345541 in HeLa cells and Vorinostat in NCI-H1299 cells. Combination treatments reduced cell viability, induced apoptosis, and promoted G2/M cell cycle arrest compared with primary drugs alone. DSG-guided transcriptional reversal offers a rational framework for overcoming therapeutic resistance. Our findings demonstrate that chaetocin can restore drug sensitivity in resistant cancer models, supporting its potential as a resistance-modulating agent in combination therapies. Given its epigenetic activity, chaetocin aligns with the emerging role of epigenetic modulators as promising partners in oncological co-treatments.

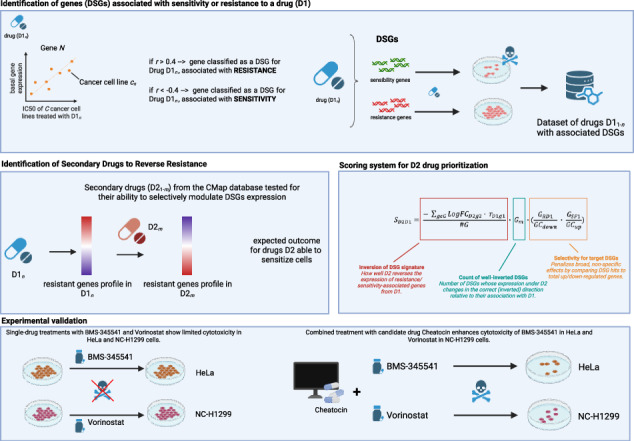

## Highlights

- A DSG-based strategy identifies compounds reversing drug resistance.
- Integration of DSGs with CMap enables rational sensitizer discovery.
- Chaetocin restores sensitivity to BMS-345541 and Vorinostat in cancer cells.
- Combination treatments trigger apoptosis and G2/M arrest in resistant models.
- Epigenetic modulators emerge as promising partners in oncology co-therapies.

## Introduction

Cancer remains a formidable challenge in medicine, with drug resistance posing one of the most significant barriers to successful treatment. Resistance mechanisms, both intrinsic, arising from pre-existing genetic or epigenetic alterations, and acquired, developing under therapeutic pressure, severely limit the efficacy of current cancer therapies. These mechanisms include aberrant expression of drug efflux transporters, activation of alternative survival pathways, and rewiring of transcriptional programs that enable cancer cells to evade drug-induced cytotoxicity [1, 2]. Overcoming these diverse and dynamic resistance mechanisms requires a shift from static, single-target approaches to more flexible and systems-level strategies capable of capturing the complexity of tumor adaptation [3]. Overcoming resistance is thus a critical objective in cancer research, as it holds the key to improving survival rates and patient outcomes [4]. Beyond germline variability, recent pharmacogenomic research has increasingly emphasized transcriptomic features as key determinants of drug response. In particular, gene expression signatures have proven to be highly predictive of sensitivity or resistance to specific compounds, often outperforming genomic alterations alone in large-scale pharmacogenomic screens [5]. Within this framework, drug-specific genes (DSGs), defined as genes whose expression levels are statistically associated with the response to a given drug across diverse cellular contexts, have emerged as critical tools for dissecting the molecular basis of pharmacological sensitivity [6]. Unlike classical pharmacogenes involved in drug metabolism (e.g., CYPs, UGTs), DSGs can reflect both upstream regulatory changes and pathway-level adaptations that mediate intrinsic or acquired resistance. Their identification, often through integrative analyses of transcriptomic and pharmacological data (e.g., GDSC, CCLE, PRISM), provides functional insights into drug mechanism-of-action and uncovers non-obvious dependencies that may serve as alternative targets for combination therapy or drug repurposing [5–7].

Moreover, recent deep learning and machine learning approaches have leveraged DSGs to enhance drug response prediction models, integrating gene expression with compound structure or target profiles to infer drug sensitivity across untested contexts.

DSGs identification in a pan-cancer context allows for the development of broad yet precise strategies to counter resistance across tumor types [8].

Despite the growing availability of multi-omics datasets and computational tools, actionable frameworks that translate pharmacogenomic findings into therapeutic strategies remain limited. In this context, the integration of DSGs with perturbational data offers a unique opportunity to rationally design drug combinations aimed at re-sensitizing resistant cancer cells. The Connectivity Map (CMap) offers a transformative resource for leveraging DSGs to address resistance [9]. By profiling gene expression changes induced by thousands of chemical and genetic perturbations, CMap enables the systematic identification of secondary drugs that modulate DSG expression. This approach has been successfully used to uncover compounds capable of reversing aberrant transcriptional signatures associated with drug resistance, thereby sensitizing cancer cells to primary therapies [9, 10]. Despite its potential, the translation of CMap findings into clinical applications requires robust scoring and ranking systems to prioritize the most effective secondary drugs while minimizing off-target genomic effects [11].

In this study, we introduce a novel pharmacogenomic framework designed to overcome drug resistance by targeting the regulatory mechanisms of DSGs. Rather than focusing solely on the direct relationship between DSG expression and drug response, our approach aims to identify secondary drugs that can modulate the expression of DSGs associated with resistance. By integrating gene expression data and drug perturbation profiles across multiple cancer cell lines, we systematically search for compounds capable of reversing the expression patterns of key DSGs, thereby sensitizing resistant cells to primary treatments. This strategy enables the discovery of combinatorial therapies that act through transcriptional reprogramming of resistance-associated genes. Secondary drugs from the CMap dataset were ranked based on their ability to selectively modulate DSG expression, employing a novel scoring algorithm that quantifies their effectiveness. We complement this computational strategy with experimental validation in selected resistant cancer cell lines, demonstrating the translational relevance and biological impact of our predictions. By integrating genomic, transcriptomic, and pharmacologic data, this work not only identifies promising candidates for combination therapies but also provides mechanistic insights into the pathways driving resistance.

## Results and discussion

### Drug-specific genes (DSGs) and their role in drug response

**Fig. 1:**
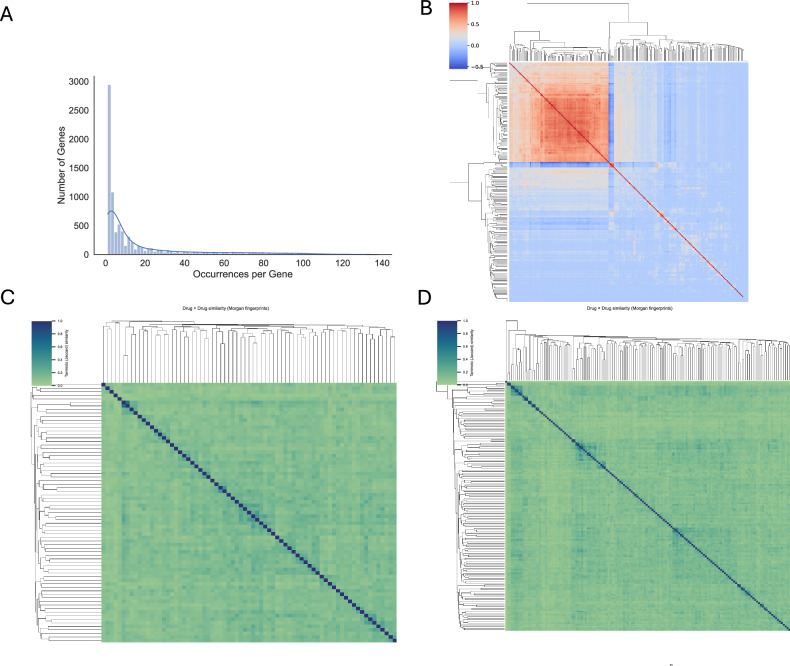
Landscape of gene–drug associations and drug similarity profiling based on DSG signatures. **A** Distribution of the number of DSGs (y-axis) as a function of the number of drugs in which each gene was identified (x-axis); **B** Drug–drug similarity heatmap based on continuous gene-level correlation profiles. Similarity was computed by clustering drugs using the full vector of DSG–drug correlation coefficients, capturing both the direction and strength of gene–drug associations and highlighting two main drug clusters; **C** Structural similarity among drugs belonging to the cluster with high DSG similarity; **D** Structural similarity among drugs belonging to the cluster with low DSG similarity.

**Fig. 2:**
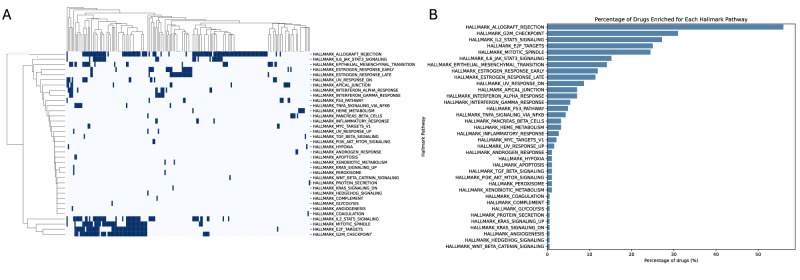
Functional enrichment and clustering of Hallmark pathways associated with Drug Sensitivity Genes. **A** Unsupervised clustering of the drug–pathway enrichment matrix derived from Hallmark gene set enrichment analysis of drug-associated DSGs; **B** Ranking of Hallmark pathways by the proportion of drugs showing significant enrichment.

We identified a set of Drug-Specific Genes (DSGs) for each drug (D1) in a pan-cancer context, as described in Parca et al. [6] and detailed in the Methods section. The analysis was based on a dataset generated by Iorio et al., which includes gene expression and drug response data (IC50) for 1001 cancer cell lines from 29 tissue types, treated with 265 drugs. Gene expression was measured using the Affymetrix Human Genome U219 Array and normalized using z-scores. For each D1, we focused on a subset of informative genes associated with drug resistance or sensitivity, inferred from their correlation with IC50 values across the cell lines. Genes positively correlated with IC50 were considered markers of resistance, while those negatively correlated were linked to sensitivity. By applying this approach to 17,419 protein-coding genes, we identified an average of 534 DSGs per drug, providing insight into cellular mechanisms of drug response beyond known drug targets. In total, across all 265 drugs, we detected 8454 unique DSGs, of which 1963 were specific for a single drug (Fig. 1A). To systematically compare these gene sets, we calculated pairwise similarities using the Tanimoto coefficient, which revealed two major groups: 91 drugs sharing a similarity ≥ 0.4, and 174 drugs displaying largely distinct profiles. This clustering pattern is clearly reflected in the drug-by-drug similarity heatmap, where two main clusters emerge (Sup Fig. 1). It should be noted that the Tanimoto coefficient was used here to cluster drugs based on the extent of overlap among their associated DSG sets, thereby quantifying how many resistance- or sensitivity-linked genes are shared or distinct between drugs, independently of their effect size or direction. To complement this set-based comparison, we additionally performed a correlation-based similarity analysis that captures the direction and magnitude of gene–drug associations. Compared to the Tanimoto-based analysis, clustering based on DSG–drug correlation profiles highlighted stronger functional similarity among epigenetic modulators (e.g., Vorinostat and Tubastatin A) and among classical chemotherapeutic agents, indicating that these compounds induce comparable resistance-associated transcriptional programs despite differences in chemical structure or target specificity (Fig. 1B). We next compared the structural similarities of the drugs within the two clusters to assess whether the overlap in DSGs was associated with chemical similarity. This analysis revealed no relationship between drug structure and gene response, as neither the cluster with highly similar DSG profiles (Fig. 1C) nor the cluster with largely distinct profiles (Fig. 1D) showed evidence of structural similarity among the drugs. To provide a biological context to the DSGs, we performed Hallmark-based gene set enrichment analysis for each drug-associated DSG list. Integration of enrichment results across compounds revealed recurrent biological programs associated with drug response. Unsupervised clustering of a drug–pathway enrichment matrix (Fig. 2A) and ranking of pathways by the proportion of enriched drugs (Fig. 2B) consistently highlighted cell cycle control, immune and inflammatory signaling, and epithelial–mesenchymal transition as dominant themes. These findings indicate that DSGs capture shared transcriptional states linked to pharmacological sensitivity and resistance, providing functional support for the proposed framework.

### Identification of secondary drugs to reverse resistance

To identify compounds capable of reversing resistance to a given treatment, we used the Connectivity Map (CMap), a large-scale resource of gene expression profiles from human cancer cell lines treated with thousands of small molecules and genetic perturbations. Starting from the DSGs identified for each primary drug (D1), we searched for secondary drugs (D2) that are able to modulate such DSGs. We hypothesized that such drugs could overcome resistance by reversing the gene expression patterns associated with reduced sensitivity to D1. To this end, we investigated whether pre-treatment with a second drug (D2) could re-sensitize resistant cell lines to D1. We analyzed CMap perturbation signatures and ranked D2 candidates according to their ability to invert the expression profiles of D1-associated DSGs. To prioritize the most relevant compounds, we implemented a scoring algorithm that favored D2 candidates selectively modulating DSGs while minimizing off-target genomic effects. These scores were subsequently transformed into z-scores to improve statistical robustness, enabling the identification of the most promising pre-treatment agents.

The scoring formula is as follows:

$${S}_{D2D1}=\frac{-{\sum }_{g\in G}{{LogFC}}_{D2g}\cdot {r}_{D1g}}{{\rm{\#}}\,G}\cdot {G}_{m}\cdot \left(\frac{{G}_{{RD}1}}{{{GC}}_{{down}}}\cdot \frac{{G}_{{SD}1}}{{{GC}}_{{up}}}\right)$$

Where:

- S_D2D1_: The score for D2 indicates its effectiveness in modifying the DSGs of D1.
- LogFC_D2,g_: Logarithmic fold change in the expression level of gene g after treatment with D2, compared to its baseline expression level.
- r_D1,g_: Pearson correlation coefficient between the basal expression profile of gene g and the IC50 profile of D1, indicating the gene’s association with sensitivity (negative r) or resistance (positive r) to D1.
- G: Set of vectors containing DSGs.
- G_m_: Number of DSGs well-inverted by D2.
- #G: Cardinality of G.
- G_R, D1_: Number of DSGs resistant to D1, downregulated by D2.
- G_S, D1_: Number of DSGs sensitive to D1, upregulated by D2.
- G_C, up_: Number of genes upregulated by D2.
- G_C, down_: Number of genes downregulated by D2.

This strategy enabled us to identify candidate compounds with potential to enhance the efficacy of primary therapies by targeting underlying resistance mechanisms.

### Secondary drugs and the role of chaetocinin sensitizing resistant cell lines

**Fig. 3:**
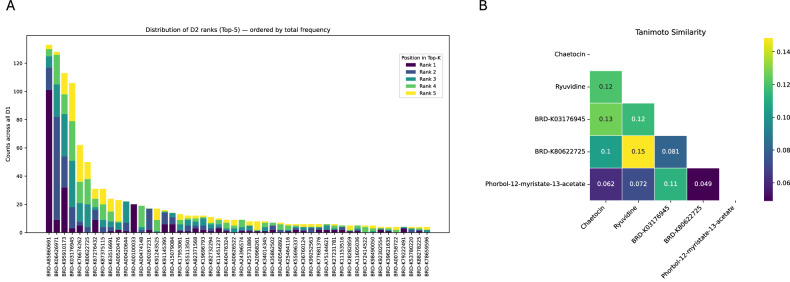
Identification and structural characterization of candidate secondary drugs for inverting Drug-Specific Gene (DSG) expression profiles. **A** Frequency of secondary drugs (D2) appearing among the top five inferred candidates for inverting the expression profile of Drug-Specific Genes (DSGs) associated with primary drugs (D1). The y-axis indicates how many times each D2 was ranked in the top 5 across all D1s analyzed. Common drug names are shown when available. Bar colors denote the position of D2 within the top-5 ranking: dark purple = Rank 1, blue = Rank 2, green = Rank 3, light green = Rank 4, yellow = Rank 5; **B** Tanimoto similarity heatmap of secondary drugs reversing resistance in at least 10 primary therapies. Pairwise Tanimoto similarity scores were computed using SMILES representations to assess the structural similarity among the most effective secondary drugs.

For each primary drug in the GDSC dataset (see “Methods”), we ranked secondary drugs from the Connectivity Map (CMap) dataset based on their ability to restore sensitivity in resistant cancer cell lines. This evaluation assessed the ability of each secondary compound to effectively reverse resistance-associated expression profiles linked to the primary drug. To obtain a global overview, we selected the top five secondary drugs (D2) for each primary drug (D1), and summarized the distribution of the 50 most frequently recurring D2 in Fig. 3A. This analysis revealed that four compounds exert a broad-spectrum effect, being selected among the top five candidates for more than 100 different D1. Among them, BRD-A85860691 (Chaetocin) demonstrated the highest sensitization score for approximately 120 primary drugs, underscoring its potential as a broad-spectrum sensitizer. Other notable compounds included BRD-K06426971 (Ryuvidine), BRD-K95910173, and BRD-03176945, each showing significant efficacy in reversing resistance for at least 100 primary drugs. In order to evaluate whether the observed activity of these secondary drugs might be explained by structural similarity, we compared the chemical structures of the compounds that reversed resistance for at least 10 primary drugs. The comparison was performed using SMILES strings and Tanimoto similarity scores. Using SMILES strings and Tanimoto similarity scores, we found low pairwise similarity values (ranging from 0.049 to 0.15), as shown in Fig. 3B. This suggests that the compounds are structurally diverse. Furthermore, their mechanisms of action also differ substantially: BRD-A85860691 (Chaetocin) targets the histone methyltransferase SUV39H1; BRD-K06426971 (Ryuvidine) inhibits DNA topoisomerase II; BRD-K80622725 interferes with RNA synthesis via RNA polymerase inhibition; and BRD-K91145395 and BRD-A15079084 function as protein kinase C (PKC) activators. These differences support the idea that the ability to reverse resistance is not due to common structural or target features, but rather to convergent phenotypic outcomes through distinct biological pathways.

### Efficacy of Chaetocin in reversing resistance profiles in resistant cell lines

Given the predicted ability of chaetocin to sensitize the highest number of primary drug-resistant cell lines among the analyzed compounds, we selected it for further investigation. Chaetocin treatment could potentially act by upregulating sensitivity-associated DSGs and downregulating resistance-associated DSGs, as observed across more than 120 primary drugs in our computational analysis. To validate this result, we tested chaetocin on two distinct cell lines: HeLa cells, which were already present in the GDSC1 dataset used for DSG identification, and NC-H1299 cells, which were absent from GDSC1, but were included in an updated GDSC2 dataset and can thus be used for independent validation. HeLa cells were specifically chosen due to their resistance to BMS-345541 (an IKK inhibitor), and NC-H1299 cells for their resistance to Vorinostat (a histone deacetylase inhibitor).

**Fig. 4:**
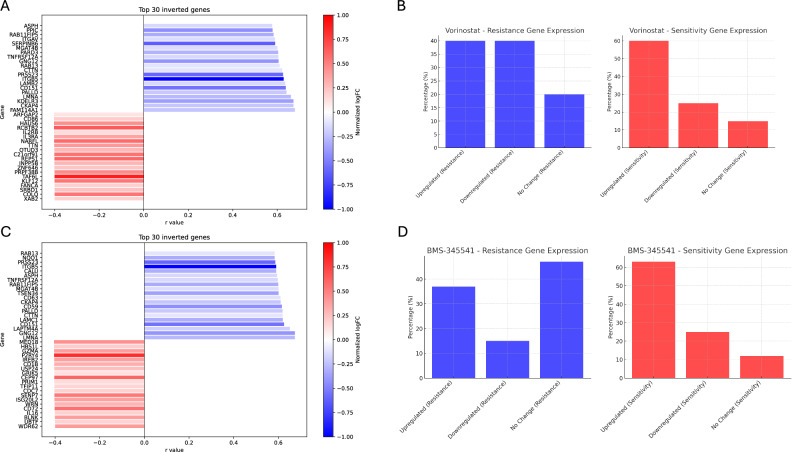
Modulation of differentially sensitive genes (DSGs) by Chaetocin to restore drug-sensitive expression profiles. Chaetocin reverses gene expression patterns associated with resistance to Vorinostat (**A**) and BMS-345541 (**C**). The figure displays the top 30 differentially sensitive genes (DSGs) following chaetocin treatment. Genes previously linked to sensitivity are upregulated (blue), while those associated with resistance are downregulated (red), reflecting a shift toward a drug-sensitive transcriptional profile; Proportion of DSGs that were upregulated, downregulated, or unchanged in response to chaetocin for Vorinostat (**B**) and BMS-345541 (**D**).

The DSG analysis for Vorinostat revealed 484 genes associated with resistance and 876 associated with sensitivity. Of the resistance-related genes, approximately 40% were downregulated by chaetocin, and around 20% showed no change in their transcriptional profile. For sensitivity-associated genes, about 60% were upregulated, and 25% remained unaffected by the treatment (Fig. 4B). In the case of BMS-345541, 440 resistance-associated genes and 757 sensitivity-associated genes were identified. For the resistance genes, only 37% were downregulated by chaetocin, and 15% did not exhibit any change. With respect to sensitivity-associated genes, 63% were upregulated, while 25% showed no transcriptional change (Fig. 4D). Figure 4A–C illustrate the fold changes in the top 30 differentially sensitive genes (DSGs) following chaetocin treatment, highlighting a consistent pattern in which sensitivity-associated genes are significantly upregulated, while resistance-associated genes are markedly downregulated, for Vorinostat and BMS-345541, respectively. Notably, this inverted expression trend aligns with the expected therapeutic direction: genes that correlate positively with drug sensitivity increase in expression, whereas resistance-associated genes decrease. This observation reinforces the hypothesis that chaetocin effectively reprograms the transcriptomic profile toward a more drug-sensitive state, supporting its role as a potent and broadly applicable sensitizing agent across both computational frameworks tested.

### The combination of Cheatocin with Vorinostat or BMS-345541 significantly reduces cell viability in both NCI-H1299 and HeLa cells

**Fig. 5:**
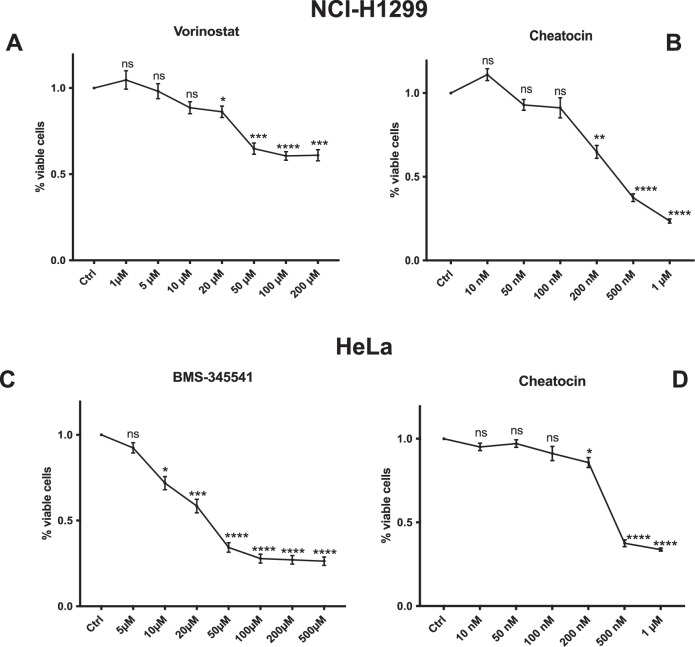
Dose-response analysis of Vorinostat, BMS-345541, and Cheatocin in NCI-H1299 and HeLa cells. NCI-H1299 cells were treated for 24 h with increasing concentrations of Vorinostat (**A**) and Cheatocin (**B**), while HeLa cells were exposed to BMS-345541 (**C**) and Cheatocin (**D**). Cell viability was assessed using the MTT assay for both cell lines. Bar column represents mean ± S.E.M.; statistical significance was determined using one-way analysis of variance (ANOVA). (ns *p* > 0.05; *p < 0.05; **p < 0.01; *** *p* < 0.001; ****p < 0.0001). All statistical analyses were performed using Prism 10 (GraphPad).

To identify suitable working concentrations for downstream experiments, we first assessed the dose-response curves of NCI-H1299 and HeLa cell lines to Vorinostat, BMS-345541, and Cheatocin. Based on IC₅₀ values from the Cell Model Passport database (Van der Meer et al., 2019), Vorinostat has an IC₅₀ of 1567 μM in NCI-H1299, and BMS-345541 has an IC₅₀ of 7192 μM in HeLa cells. Cell viability was assessed after 24 h of treatment using the MTT assay. In NCI-H1299 cells, Vorinostat at 1, 5 and 10 µM did not induce a statistically significant reduction in viability after 24 h of treatment (Fig. 5A). c In HeLa cells, increasing concentrations of BMS-345541 (5, 10, and 20 µM) induced less than 50% reduction in viability, with 5 µM showing no statistically significant difference compared to the untreated control (Fig. 5C). A working concentration of 5 µM was selected for both cell lines, as it remained below the IC₅₀ in both cases and exhibited minimal baseline toxicity. We next evaluated the dose–response of NCI-H1299 and HeLa cell lines to Cheatocin. As shown in Fig. 5B, D, MTT assay results did not reveal a significant reduction in cell viability at 10, 50, or 100 nM in either cell line. However, 200 nM was associated with a marked cytotoxic effect. Based on these observations, 100 nM was considered an appropriate concentration for further experiments, as it represented the highest dose without a statistically significant impact on viability compared to untreated controls.

**Fig. 6:**
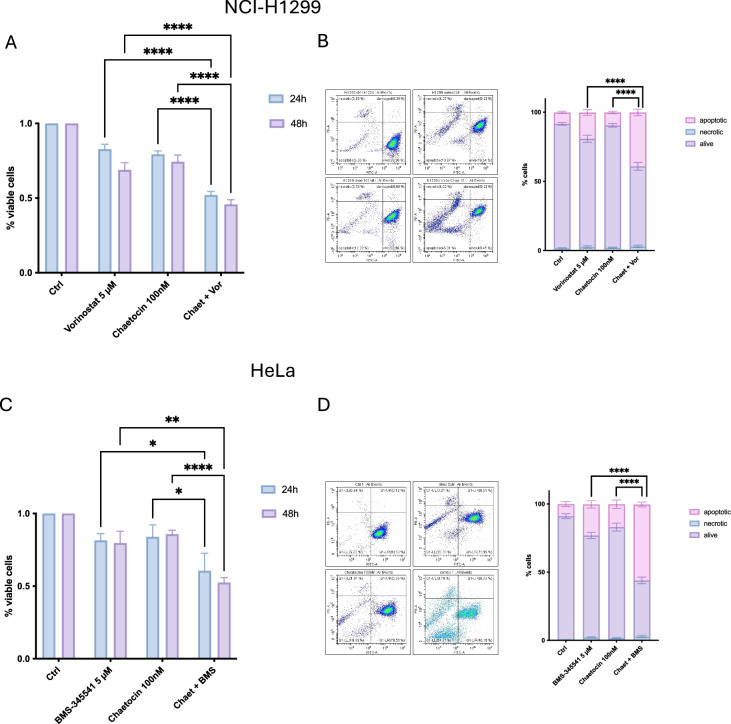
Combined treatment with Cheatocin and Vorinostat or BMS-345541 reduces cell viability and promotes apoptosis in NCI-H1299 and HeLa cells. NCI-H1299 cells were pre-treated with Cheatocin (100 nM) for 3 h, followed by Vorinostat (5 µM) for 24 h. HeLa cells were co-treated for 24 h with Cheatocin (100 nM) and BMS-345541 (5 µM). **A**, **C** Cell viability was assessed by MTT assay at 24 and 48 h. **B**, **D** FDA/PI staining confirmed that the combined treatments significantly reduced cell viability and induced apoptotic cell death. The bars represent mean ± S.E.M.; statistical significance was determined using one-way analysis of variance (**A**, **C**) or two-way analysis of variance (**B**, **D**) (ANOVA). (*p < 0.05; ****p < 0.0001). All statistical analyses were performed using Prism 10 (GraphPad).

We then investigated the effects of Cheatocin in combination with either Vorinostat or BMS-345541 on NCI-H1299 and HeLa cells by treating the cells with the respective drugs for 24 h, followed by an MTT assay. Our results showed that treatment with either Cheatocin or the drug that the cell line is sensitive to (Vorinostat for NCI-H1299, BMS-345541 for HeLa) alone resulted in a slight reduction in cell viability. In contrast, the combination of Cheatocin with Vorinostat in NCI-H1299 cells or Cheatocin with BMS-345541 in HeLa cells led to a significant decrease in cell viability, at both 24 and 48 h after treatment, suggesting a synergistic effect as predicted by the computational analysis. To further validate the MTT results, we performed fluorescein diacetate (FDA) and propidium iodide (PI) viability assays to distinguish between apoptosis and necrosis. Cells were treated using the same experimental procedure as for MTT. The results were consistent with those observed in the MTT assay. Treatment with either Cheatocin, or Vorinostat, or BMS-345541 alone resulted in a moderate reduction in cell viability, as shown in Fig. 6. However, the combination of the two drugs induced a significant reduction in the number of viable cells after 24 h. Importantly, cell death induced by the combined treatment was predominantly apoptotic rather than necrotic, as confirmed by the FDA/PI assay. These results strongly support the hypothesis that the combination of Cheatocin with Vorinostat or BMS-345541 enhances their pro-apoptotic effects and significantly increases their cytotoxicity in both NCI-H1299 cells and HeLa cells. To assess the specificity of the computational predictions, we performed a negative experimental validation using Doxorubicin in NCI-H1299 cells, a model known to be resistant to this drug (Supplementary Fig. 2A). Consistent with pharmacogenomic annotations, chaetocin ranked poorly among compounds predicted to reverse doxorubicin resistance. Experimentally, chaetocin pre-treatment did not enhance doxorubicin sensitivity (Supplementary Fig. 2B), confirming the negative prediction and supporting the discriminative power of the computational framework.

### Combined treatment with Cheatocin and Vorinostat or BMS-345541 induces G2/M cell cycle arrest in NCI-H1299 and HeLa cells

**Fig. 7:**
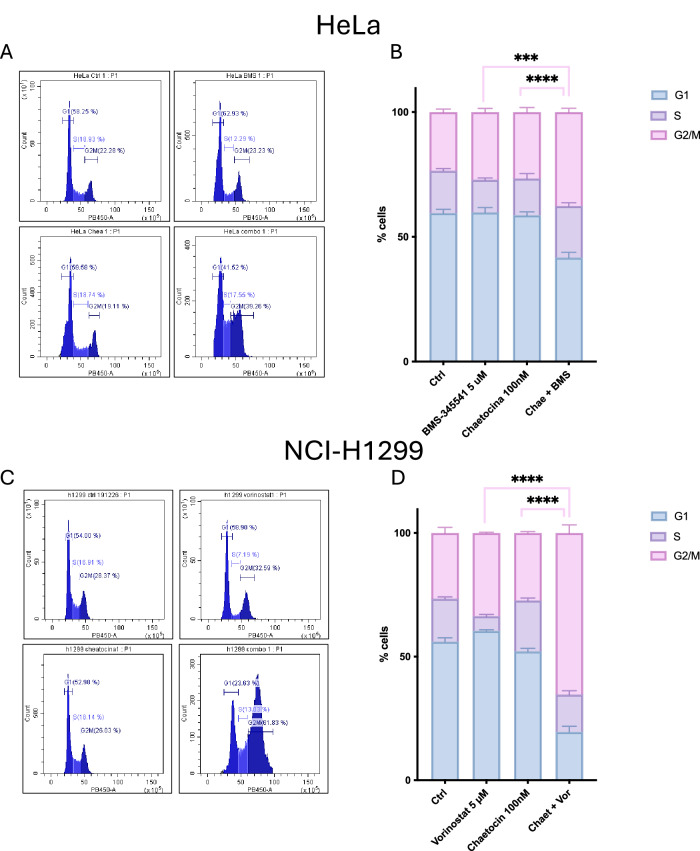
Chaetocin promotes G2/M phase accumulation and G1 depletion in combination therapies. Cheatocin combined with Vorinostat (**C**) or BMS-345541 (**A**) promotes G2/M cell cycle arrest in NCI-H1299 and HeLa Cells. The cells were treated with Cheatocin (100 nM), Vorinostat (5 µM), BMS-345541 (5 µM) or combinations of these for 24 h. Cell cycle distribution was analysed using DAPI staining and flow cytometry. In both cell lines, the proportion of cells in the G2/M phase increased significantly with combination treatment, while the G1 population decreased, indicating G2/M cell cycle arrest (**B**–**D**). The bars represent mean ± S.E.M.; statistical significance was determined using two-way analysis of variance (ANOVA). (***p < 0.001; ****p < 0.0001). All statistical analyses were performed using Prism 10 (GraphPad).

To further investigate the mechanisms underlying the cytotoxic effects of the combination treatments, we analyzed their effects on cell cycle progression in both NCI-H1299 and HeLa cells. Following the same experimental plan, we treated the cells for 24 h and then stained them with DAPI to assess cell cycle phase. In NCI-H1299 cells, treatment with Cheatocin alone did not induce significant changes in the cell cycle, while Vorinostat led to a slight increase in G2/M phase and a modest decrease in S phase. More importantly, the combination of both resulted in a strong G2/M block and a marked reduction in the G1 phase. In the same way, treatment of HeLa cells with Cheatocin or BMS alone caused limited changes in cell cycle distribution. However, co-treatment with both compounds resulted in a significant accumulation of cells in the G2/M phase and a reduction in the G1 phase (Fig. 7). This suggests that the combination treatment has a potent effect on cell cycle progression, arresting cells in the G2/M transition, which may contribute to the observed cytotoxic effects.

## Discussion

In this study, we developed and validated a novel integrative strategy to tackle one of the most pressing challenges in oncology: resistance to anticancer therapies. By exploiting the power of pharmacogenomic data, we identified Drug-Specific Genes (DSGs) as transcriptional biomarkers capable of capturing key mechanisms underlying drug sensitivity and resistance across a broad panel of cancer cell lines. Whereas traditional pharmacogenomic approaches usually focus on the expression or mutation status of the direct target of a drug to explain sensitivity, our framework instead prioritizes genes whose basal expression profiles correlate significantly with drug response, regardless of whether they represent canonical targets. This strategy provides a more systemic and unbiased view of resistance-associated molecular programs, potentially uncovering alternative pathways that modulate therapeutic efficacy.

Building upon this foundation, we implemented a rational approach to identify secondary drugs capable of reversing resistance-associated transcriptional signatures. Using the Connectivity Map (CMap), we screened thousands of drug perturbation profiles and uniquely integrated them with our DSG framework. This integration represents the main novelty of our work, as it combines resistance-specific transcriptional biomarkers with large-scale perturbation data to systematically prioritize compounds predicted to resensitize resistant cells. To this end, we developed a robust scoring system that quantitatively evaluates the ability of each candidate to selectively revert the expression of both resistance- and sensitivity-associated DSGs.

Among the compounds we analyzed, chaetocin emerged as a potent sensitizer, capable of restoring responsiveness to a broad spectrum of primary drugs. Experimental validation confirmed that chaetocin significantly enhances the cytotoxicity of Vorinostat and BMS-345541 in resistant NCI-H1299 and HeLa cells, respectively. The combination treatments not only reduced cell viability more effectively than single agents, but also induced apoptotic cell death and G2/M cell cycle arrest, supporting the mechanistic relevance of our in silico predictions. The strong sensitizing effect of chaetocin is particularly intriguing in light of its epigenetic activity as a histone methyltransferase inhibitor. Epigenetic modulators, including inhibitors of histone deacetylases (HDACs), DNA methyltransferases (DNMTs), and histone methyltransferases (HMTs), have long been investigated as adjuvants to conventional chemotherapies. A large body of evidence demonstrates that such agents can reprogram chromatin accessibility and transcriptional networks, thereby restoring apoptotic responses, enhancing DNA damage, and overcoming multidrug resistance phenotypes [12–14]. For instance, HDAC inhibitors such as vorinostat and panobinostat have been shown to synergize with platinum compounds and topoisomerase inhibitors, while DNMT inhibitors like azacitidine or decitabine can resensitize tumors to cytotoxic agents by reversing epigenetically silenced tumor suppressor pathways [15, 16]. More recently, HMT inhibitors have attracted interest for their ability to rewire oncogenic gene expression programs and augment chemotherapy efficacy [17, 18]. Our findings with chaetocin align with this literature, supporting the broader concept that epigenetic therapy can be leveraged to perturb resistance-associated transcriptional states and enhance the therapeutic impact of standard chemotherapies.

These findings support the notion that targeting cellular states associated with drug resistance may represent a powerful and generalizable strategy to enhance therapeutic efficacy. By shifting the focus from single-gene alterations to coordinated gene expression programs, our approach provides a framework that can be adapted to a wide variety of tumor types and therapeutic contexts. Furthermore, the identification of broadly effective sensitizers like chaetocin opens new avenues for the development of combination therapies aimed at overcoming both intrinsic and acquired resistance. Future studies in patient-derived models and in vivo systems will be essential to validate these findings and to assess their clinical translatability. Importantly, the identification of effective sensitizing combinations involving drugs not conventionally used in these cancer types supports a drug repurposing strategy, highlighting how resistance-associated transcriptional states and pathway-level vulnerabilities can be exploited independently of existing treatment paradigms.

## Materials and methods

### Data collection

The pharmacogenomic dataset used in this study was generated in a foundational work by Iorio et al. [5], representing an expansion of earlier datasets [7, 8]. This dataset, accessible through the Genomics of Drug Sensitivity in Cancer (GDSC) portal (https://www.cancerrxgene.org/gdsc1000/GDSC1000_WebResources/Home.html), includes pre-treatment gene expression profiles for 17,419 genes across 1001 cancer cell lines derived from 30 tumor tissues. Gene expression profiling was conducted using the [HG-U219] Affymetrix Human Genome U219 Array (A-GEOD-13667). Beyond transcriptional profiling, the dataset provides extensive genomic, epigenetic, and pharmacological characterizations.

Drug sensitivity data for 265 compounds, spanning clinical, developmental, and experimental categories, were included, with sensitivity quantified by IC50 values, a measure of the drug concentration required to inhibit 50% of cell viability in vitro. The compounds encompass 19 cytotoxic agents and 242 targeted therapies, addressing 20 key pathways and processes central to cancer biology.

### Connectivity Map

Connectivity Map (CMap), a database comprising transcriptional response signatures to perturbagens, was utilized to identify drugs capable of modulating expression profiles of drug-specific genes (DSGs). Perturbagens were applied to cell lines under standard conditions (10 µM dose, 24-h exposure), and expression data were analyzed using GCTX files with the cmapPy Python package.

### Identification of drug-specific genes (DSGs)

For each primary drug (D1), the genes associated with sensitivity or resistance were identified using the methodology proposed by Parca et al. [6]. Pearson correlation analysis was applied to assess the relationship between basal gene expression profiles and IC50 values across 962 shared cancer cell lines. Genes exhibiting a Pearson correlation coefficient of ≥ +0.4 or ≤ –0.4 were classified as Drug-Specific Genes (DSGs), indicating a significant association with drug sensitivity or resistance.

To enhance the specificity of these associations, the analysis was further refined by focusing on the top and bottom 20% of cell lines that displayed extreme expression levels for each gene. This targeted selection highlighted the most informative relationships between gene expression and drug response, providing a robust framework for subsequent analyses.

### Mathematical definition of the response-informed score

For each gene *g*, we computed the Pearson correlation coefficient *r*^D1,*g*^ between its basal expression profile and the IC50 values of the reference drug D1, so that positive correlations indicate association with resistance and negative correlations indicate association with sensitivity to D1. Let *G* denote the set of differentially expressed genes (DSGs) associated with the response to D1, and #*G* its cardinality.

For each candidate compound D2, we obtained the log fold-change in expression of each gene *g* ∈ *G* after treatment with D2, denoted logFC^D2,*g*^. The response-informed score *S*_D2D1_ that quantifies the ability of D2 to modulate the DSGs of D1 is defined as:

$${S}_{D2D1}=\frac{-{\sum }_{g\in G}{{LogFC}}_{D2g}\cdot {r}_{D1g}}{{\rm{\#}}\,G}\cdot {G}_{m}\cdot \left(\frac{{G}_{{RD}1}}{{{GC}}_{{down}}}\cdot \frac{{G}_{{SD}1}}{{{GC}}_{{up}}}\right)$$

Here, *G*_*m*_ is the number of DSGs that are inverted by D2 in a way that promotes sensitivity to D1, that is, resistance-associated genes (with *r*^D1,g^ > 0) that are downregulated by D2 and sensitivity-associated genes (with *r*^D1,g^ < 0) that are upregulated by D2. *G*^R,D1^ is the number of DSGs associated with resistance to D1 that are downregulated by D2, and *G*^S,D1^ is the number of DSGs associated with sensitivity to D1 that are upregulated by D2. *G*^*C,up*^ and *G*^*C,down*^ denote, respectively, the total number of genes upregulated and downregulated by D2 in the corresponding perturbation profile. The multiplicative term

$${G}_{m}\cdot \left(\frac{{G}_{{RD}1}}{{{GC}}_{{down}}}\cdot \frac{{G}_{{SD}1}}{{{GC}}_{{up}}}\right)$$

Therefore emphasizes compounds that selectively invert a large fraction of resistance and sensitivity DSGs while controlling for the overall breadth of transcriptional changes induced by D2, thus penalizing non-specific perturbations.

In this formulation, higher values of *S*_D2D1_ indicate compounds that, on average, decrease the expression of resistance-associated genes and increase the expression of sensitivity-associated genes, with each gene weighted by the strength and direction of its correlation with the response to D1.

### Cell culture and treatment conditions

The human non-small cell lung carcinoma cell line NCI-H1299 and HeLa cells were maintained in DMEM (SIAL-DMEM-HPXA), supplemented with 10% fetal bovine serum (FBS) (yourSIAL-FBS-SA), 100 U/mL penicillin and 100 mg/mL streptomycin (#P0781), and 2 mM L-glutamine (Euroclone #ECB3000D). Cell cultures were incubated at 37 °C with 5% CO₂. The experimental conditions for NCI-H1299 cells included: (1) pretreatment with Cheatocin (100 nM) for 3 h, followed by replacement of the medium with fresh, drug-free medium; (2) treatment with Vorinostat alone (5 μM for 24 h); and (3) combination treatment consisting of a 3-h pretreatment with Cheatocin (100 nM), followed by replacement of the medium and exposure to Vorinostat (5 μM) for 24 h. For HeLa cells, the experimental setup consisted of: (1) treatment with 100 nM Cheatocin alone; (2) treatment with 5 μM BMS-345541 alone; and (3) co-treatment with 100 nM Cheatocin and 5 μM BMS-345541 for 24 h.

### MTT assay

Cells were seeded in 96-well plates at a density of 5000 cells per well in 100 µL growth medium. After treatment, cell viability was assessed using the MTT assay. MTT reagent (3-(4,5-dimethylthiazol-2-yl)-2,5-diphenyltetrazolium bromide) was prepared by dissolving the powder in distilled water at a concentration of 5 mg/mL. MTT was diluted 1:10 directly in the cell culture medium by adding 10 µL MTT solution to 100 µL medium already in each well. The plates were incubated at 37 °C for 4 h to allow formazan crystals to form, which were then dissolved in 100 µL of dimethyl sulfoxide (DMSO). Absorbance was measured at 570 nm using a microplate reader (Biorad iMark microplate reader).

### Fluorescein diacetate (FDA) and propidium iodide (PI) viability assay

Cell viability was assessed using fluorescein diacetate (FDA) and propidium iodide (PI) staining, a method that allows the differentiation of viable from non-viable cells based on fluorescence [19]. FDA, a cell-permeant esterase substrate, is hydrolyzed by intracellular esterases in viable cells, producing green fluorescence. In contrast, PI, a non-permeable nucleic acid stain, only enters cells with impaired membrane integrity and generates red fluorescence upon binding to DNA. A staining solution was freshly prepared by diluting fluorescein diacetate (FDA, 100 mM stock in DMSO #S-343209) to a final concentration of 100 nM and propidium iodide (PI, 1 mg/mL stock #P3566) to a final concentration of 1 µM in DMEM medium. The staining solution was added to each cell suspension, and samples were incubated for 10 min at room temperature. Viability was assessed using a CytoFLEX flow cytometer (Beckman Coulter). A total of 10,000 events per sample were acquired. Viable cells were identified by green fluorescence from FDA hydrolysis (FITC channel), while non-viable cells were detected by red fluorescence from PI binding (PE channel). Apoptotic cells were quantified as double negative for FDA and PI fluorescence. Data analysis was performed using CytoExpert software.

### Cell cycle profiling

For classical cell cycle analysis after treatment, the cells were collected, washed twice with phosphate-buffered saline (PBS) and fixed in 70% ice-cold ethanol overnight at –20 °C. After fixation, cells were washed again with PBS to remove residual ethanol. For DNA staining, cells were incubated with a staining solution containing 1 µg/mL 4’,6-diamidino-2-phenylindole (DAPI, # 62248) and 100 µg/mL RNase A (#12091-021) in PBS for 30 min at room temperature in the dark. After incubation, cell cycle distribution was analysed by flow cytometry (CytoFLEX). A total of 10,000 events were acquired within the P1 gate, and data were analyzed using CytoExpert software.

### Reagents

Propidium iodide (PI) (Catalog #P3566) was obtained from Thermo Fisher Scientific. Fluorescein Diacetate (FDA) (Catalog #S-343209) was purchased from Sigma-Aldrich. DAPI (Catalog #62248) and RNase A (Catalog #12091-021) were obtained from Thermo Fisher Scientific. Cheatocin (Catalog #S8068) was dissolved in Dimethyl Sulfoxide (DMSO) at a concentration of 10 mM. Vorinostat (SAHA) (Catalog #S1047) was dissolved in DMSO at a concentration of 50 mM, and BMS-345541(Catalog #S8044) was dissolved in DMSO at a concentration of 10 mM for experimental use.

## Supplementary information

Supplementary Material Legends

Supplementary Figure 1

Supplementary Figure 2

## Data Availability

Pharmacogenomic and perturbational datasets are publicly available from the Connectivity Map (CMap). Experimental data generated during this study are available from the corresponding author on request.
