# Supplementary material for: Leveraging drug-specific genes to identify sensitizers for resistant cancer cell lines
Source: Cell Death Discov. 2026 Apr 7;12:238. doi: 10.1038/s41420-026-03033-x (PMC13187434; doi:10.1038/s41420-026-03033-x)
Supplement: Supplementary file 1 — Supplementary Material Legends [file 41420_2026_3033_MOESM1_ESM.docx]

Sup Figure 1 Drug–drug similarity heatmap based on the Tanimoto coefficient calculated from DSG overlap, highlighting two main clusters of drugs.

Sup Figure 2 (a) Dose-response analysis of doxorubicin in NCI-H1299 cells. NCI-H1299 cells were treated for 24 hours with increasing concentrations of doxorubicin. Cell viability was measured using the MTT assay. Data points represent mean ± S.E.M. Statistical significance was determined by one-way ANOVA (ns, p > 0.05; **p < 0.01). All analyses were performed using GraphPad Prism 10; (b) Effect of Cheatocin pre-treatment on doxorubicin-induced cell viability in NCI-H1299 cells. NCI-H1299 cells were pre-treated with Cheatocin (100 nM) for 3 hours, followed by treatment with doxorubicin for 24 hours. Cell viability was assessed by MTT assay at 24 hours. The combination of Cheatocin pre-treatment with doxorubicin did not further reduce cell viability compared to doxorubicin alone. Data represent mean ± S.E.M. of at least three independent experiments. Statistical significance was determined by one-way ANOVA (ns, p > 0.05).
