# Supplementary figures and images for: Leveraging drug-specific genes to identify sensitizers for resistant cancer cell lines
Source: Cell Death Discov. 2026 Apr 7;12:238. doi: 10.1038/s41420-026-03033-x (PMC13187434; doi:10.1038/s41420-026-03033-x)

Drug × Drug Clustermap (Jaccard similarity)

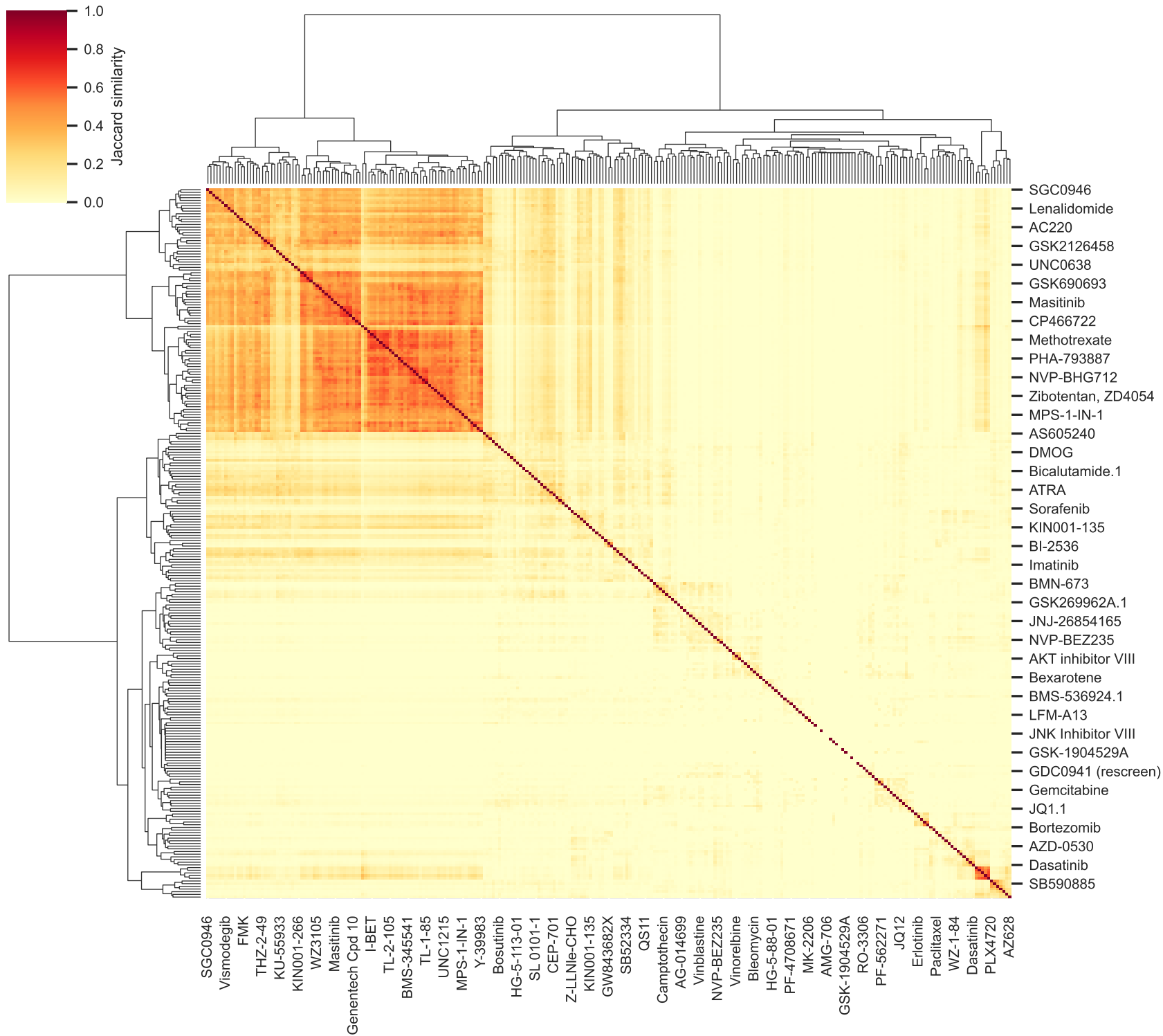

Supplement: Supplementary file 2 — Supplementary Figure 1 [file 41420_2026_3033_MOESM2_ESM.pdf]

A

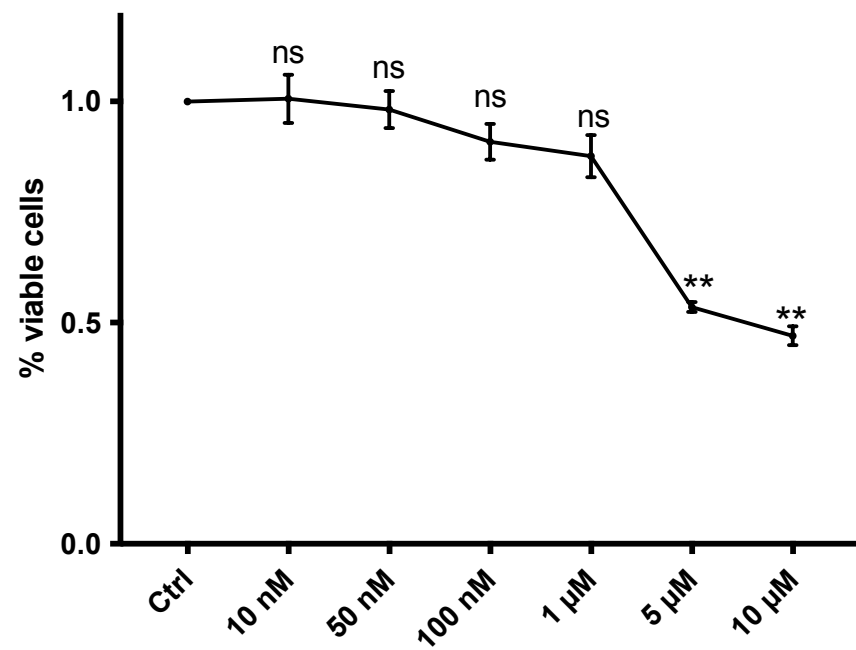

B

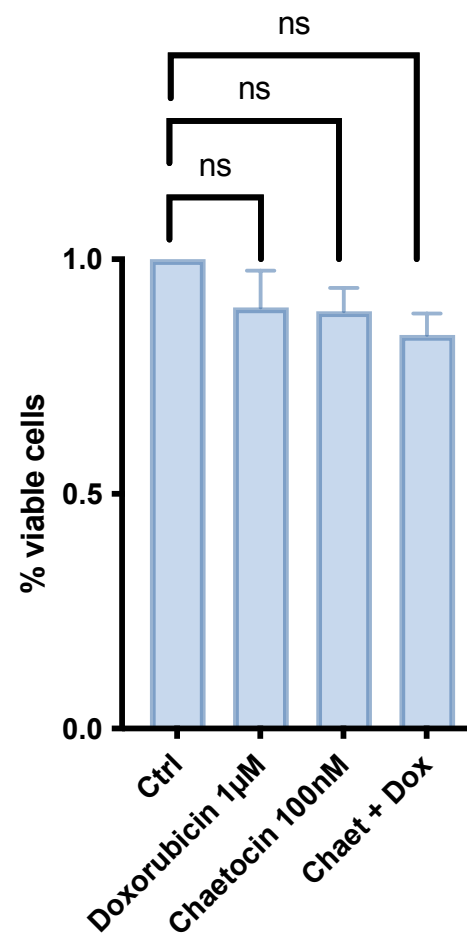

Supplement: Supplementary file 3 — Supplementary Figure 2 [file 41420_2026_3033_MOESM3_ESM.pdf]
